# Side effects of monaural beat stimulation during sustained mental work on mind wandering and performance measures

**DOI:** 10.3389/fpsyg.2024.1375717

**Published:** 2024-04-19

**Authors:** Lucy Marlene Schmidt, Leila Chaieb, Marlene Derner, Thomas P. Reber, Juergen Fell

**Affiliations:** ^1^Department of Epileptology, University Hospital Bonn, Bonn, Germany; ^2^Faculty of Psychology, UniDistance Suisse, Brig, Switzerland

**Keywords:** mind wandering, monaural beat, auditory beat stimulation, sustained attention to response task, mind wandering questionnaire, meta-awareness

## Abstract

Excessive mind wandering (MW) contributes to the development and maintenance of psychiatric disorders. Previous studies have suggested that auditory beat stimulation may represent a method enabling a reduction of MW. However, little is known about how different auditory stimulation conditions are subjectively perceived and whether this perception is in turn related to changes in subjective states, behavioral measures of attention and MW. In the present study, we therefore investigated MW under auditory beat stimulation and control conditions using experience sampling during a sustained attention to response task (SART). The subjective perception of the stimulation conditions, as well as changes in anxiety, stress and negative mood after versus before stimulation were assessed via visual-analog scales. Results showed that any auditory stimulation applied during the SART was perceived as more distracting, disturbing, uncomfortable and tiring than silence and was related to more pronounced increases of stress and negative mood. Importantly, the perception of the auditory conditions as disturbing was directly correlated with MW propensity. Additionally, distracting, disturbing and uncomfortable perceptions predicted negative mood. In turn, negative mood was inversely correlated with response accuracy for target stimuli, a behavioral indicator of MW. In summary, our data show that MW and attentional performance are affected by the adverse perception of auditory stimulation, and that this influence may be mediated by changes in mood.

## Introduction

In recent years, the phenomenon of mind wandering (MW), i.e., mental deviation from the current situation and activity (Smallwood and Schooler, 2015), has attracted increasing research interest. Initially, this research was mainly located within the fields of experimental psychology and cognitive neuroscience (Smallwood and Schooler, 2015; Christoff et al., 2016). Currently, however, an increasing number of clinical studies are focussing on the role of MW in psychiatric disorders (for overviews, see, e.g., Bozhilova et al., 2018; Chaieb et al., 2022a; Fell et al., 2023). Here, the newer approach of investigating overall MW overlaps to some extent with the preceding approaches of examining rumination and worry. Available studies suggest that the transition from common to excessive MW is a key factor contributing to the development and maintenance of psychiatric disorders, in particular attention deficit/hyperactivity disorder (Bozhilova et al., 2018), depression (Chaieb et al., 2022a), and anxiety disorders (Fell et al., 2023). This transition to excessive MW is possibly driven by several feedback loops. Most notably, the interdependences between increased MW and negative emotions (Smallwood et al., 2009; Killingsworth and Gilbert, 2010), as well as poor sleep (Zoccola et al., 2009; Cárdenas-Egúsquiza and Berntsen, 2022) may constitute vicious circles.

Therefore, the development of methods targeting a reduction of MW would be desirable. In the field of cognitive psychology, research has demonstrated that increasing participant motivation during attentional task performance reduces MW, as evaluated through experience sampling (Seli et al., 2019). Performance measures such as error rates and reaction times are commonly regarded as indicators of MW, although they often poorly correspond to experience sampling-based measures of MW (Schumann et al., 2022a). Various strategies have been shown to increase attentional task performance based on these measures, including try-harder instructions (Steinborn et al., 2016), setting specific performance goals (Strayer et al., 2023), and providing feedback based on physiological indicators of attentional lapses (De Bettencourt et al., 2015). It remains an ongoing area of research to what extent these approaches may also facilitate the reduction of task-related MW, as assessed by experience sampling-based measures (e.g., Strayer et al., 2023).

In the clinical context, one current approach to reducing MW is mindfulness training, for instance, in the context of mindfulness-based stress reduction (Ludwig and Kabat-Zinn, 2008) or mindfulness-based cognitive therapy (Segal et al., 2012). Indeed, decreases in MW and concomitant reductions in depressive and anxious symptoms due to mindfulness training have been reported (Feruglio et al., 2021; Chaieb et al., 2022a; Fell et al., 2023). However, mindfulness training requires continuous attention and diligent practice, which is beyond the capabilities of many patients, and may produce adverse effects and increase stress (e.g., Farias et al., 2016; Groves, 2016). An alternative passive option are non-invasive brain stimulation techniques, such as transcranial direct current stimulation (tDCS). So far, MW-related tDCS studies are methodologically divergent and findings are often inconclusive and even contradictory (for an overview, see Chaieb et al., 2019; Nawani et al., 2023).

Auditory beat stimulation is a novel, non-invasive and reversible brain stimulation technique (e.g., Schwarz and Taylor, 2005; Chaieb et al., 2015; Garcia-Argibay et al., 2019). A common way to generate auditory beats is to apply sine tones with slightly different frequencies. For instance, two sine tones with frequencies of 217.5 Hz and 222.5 Hz result in a beat at 5 Hz, i.e., in the theta range. The tones are either applied separately to each ear (binaural beats), or amplitude modulated signals are produced by superposition (monaural beats) and applied to one or both ears. Both application types cause a beat sensation, the former due to the activity of phase-sensitive neurons in the brain stem, the latter a result of physical acoustic properties. Although humans can only perceive tones with carrier frequencies above 16–20 Hz, they can detect amplitude-modulations with modulation frequencies as low as 1 Hz (e.g., Joris et al., 2004). According to a popular idea, binaural beats entrain EEG oscillations in the range of the modulation frequency (e.g., Schwarz and Taylor, 2005; Lavallee et al., 2011). However, only a few of the binaural beat-related studies reported EEG power increases in line with this entrainment idea, while the majority of studies found no evidence for this assumption (for an overview, see Ingendoh et al., 2023).

In a previous intracranial EEG study, we investigated the effects of both monaural and binaural beat stimulation (Becher et al., 2015). We detected a decrease of EEG power and phase synchronization in rhinal cortex and hippocampus due to monaural 5 Hz beats versus sine waves, which was absent for binaural 5 Hz beats (Becher et al., 2015). Since the hippocampus is now known to play an important role in MW (e.g., Ellamil et al., 2016; O'Callaghan et al., 2019), we hypothesized that a reduction of MW may be achievable by applying monaural beats in the theta range. Therefore, in a pilot study (Chaieb et al., 2020), we investigated the effects of auditory beat-stimulation via experience sampling of MW characteristics during a variant of the sustained attention to response task (SART; Robertson et al., 1997). We observed tentative evidence for decreased MW during monaural 5 Hz beats compared to binaural 5 Hz beats, silence, and sine wave stimulation. In a follow-up study (Chaieb et al., 2022b), monaural theta beats with shifting modulation frequencies (4–8 Hz) were employed, with subjects preselected for high MW traits based on MW questionnaire scores (Mrazek et al., 2013). Findings of this study pointed to a beat-related reduction of MW compared to sine waves and silence, in subjects with high trait levels of MW.

A yet underinvestigated but increasingly relevant topic is, how different auditory stimulation conditions are subjectively perceived, and whether this perception is related to changes in subjective states, behavioral SART measures and MW. In the present study, we therefore used visual-analog scales to assess the subjective perception of the stimulation conditions (distracting, disturbing, uncomfortable, tiring), as well as changes of anxiety, stress and negative mood after versus before stimulation. To enable evaluation of the influence of trait levels of MW (Chaieb et al., 2022b), subjects completed two well-established MW questionnaires (Mrazek et al., 2013; Mowlem et al., 2019). Aside from 5 Hz monaural beats, we also implemented a condition in which subjects were free to choose a modulation frequency in the theta range (range of choices: 3.5/4/4.5/5/5.5/6/6.5 Hz). We hypothesized that self-selected stimulation may be more positively perceived than the pre-specified stimulation conditions. Moreover, a 5 Hz monaural beat stimulation applied only to the right ear was also used, since MW has been specifically linked to the left hippocampus (Fox et al., 2015; Krakau et al., 2020) and auditory stimuli applied to one ear are processed mainly contralaterally (Woldorff et al., 1999). Furthermore, white noise was used as an additional control besides sine wave stimulation and silence, since facilitating effects of white noise stimulation on attentional and cognitive performance have been reported (e.g., Angwin et al., 2017; Awada et al., 2022). In general, we suspected that, given the requirement to continuously perform an attentional task, any auditory stimulation might be more negatively perceived than silence. Moreover, we hypothesized that adverse perception of the stimulation conditions may be correlated to unfavorable changes of subjective states, as well as increased MW and decreased SART performance.

## Materials and methods

### Participants

42 healthy subjects were recruited into the study (20 females, 10 males, 12 not specified; mean age ± s.d.: 24.4 ± 4.0 years). Subjects were recruited via printed advertisements placed within university hospital grounds, online university forums and by word of mouth. The study and all experimental procedures were approved by the Ethics Committee of the Medical Faculty of the University of Bonn, and were in accordance with the Declaration of Helsinki. All participants gave written informed consent. Participants performed the task in a cubicle, which was located in a quiet office room, and wore sound-shielded headphones.

### Self-rating mind wandering scales

All participants completed a German translation of the mind-wandering questionnaire (MWQ; Mrazek et al., 2013) and the German version of the mind excessively wandering scale (MEWS; Mowlem et al., 2019; Nakovics et al., 2021). These questionnaires are comprised of five (MWQ) and fifteen (MEWS) questions respectively, to which participants can respond using six-point (MWQ) and four-point (MEWS) Likert scales. Please refer to Mrazek et al. (2013), Mowlem et al. (2019), and Nakovics et al. (2021) for detailed information on the psychometric properties of these scales. For further analysis, data from both questionnaires were normalized by dividing by the maximum possible value, and a combined MW score was calculated thus: MWscore = (MWQ/30 + MEWS/45)/2.

### Experimental paradigm

**Figure 1:**
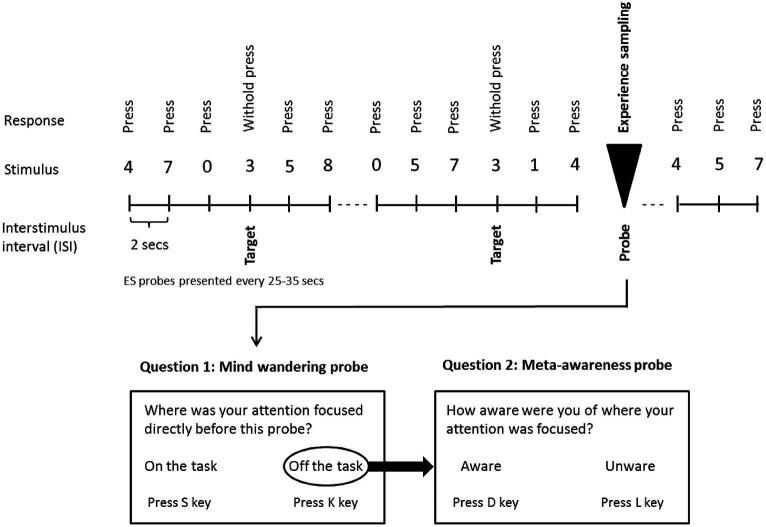
Schematic illustration of the sustained attention to response task (SART) with embedded experience sampling probes. Subjects were instructed to respond with a button press when a digit appears on the screen, with the exception of the target digit (3), for which they were instructed to withhold the button press. Subjects were also asked to respond to the embedded intermittent experience sampling probes when they appeared onscreen (Probe 1: “Where was your attention focused directly before this probe?”) (Possible responses: “on the task” or “off the task”). Whenever subjects indicated being “off-task,” a second probe assessed meta-awareness: “How aware were you of where your attention was focused?” (Possible responses: “aware” or “unaware”). Subjects were asked to respond as quickly and as accurately as possible.

Participants performed a variant of the sustained attention to response task (Robertson et al., 1997). They were asked to follow a continuous stream of digits onscreen and to press the space bar whenever a non-target number (0–2; 4–9) occurred (see Figure 1). They were further instructed to withhold the bar-press whenever the target number (3) appeared on screen. Stimuli were presented until a response was detected, or for a maximum duration of 2 s, with the inter-stimulus interval being 2 s. Participants were instructed to respond as quickly and as accurately as possible. Each participant performed six runs of the SART. Each run had a duration of approximately 15 min (i.e., responses to 25 experience sampling probes were acquired; see below), and during each run a different auditory beat stimulation condition was administered (within-subject manipulation). The order of runs was randomized and counterbalanced across subjects (see below). Between runs participants were offered a short break of approximately 5 min.

### Experience sampling

To examine the propensity to mind wander and meta-awareness of MW, experience sampling probes were embedded intermittently within the SART digit stream (see Figure 1). The first probe addressed the subject’s focus of attention immediately before appearance of the probe: “Where was your attention focused directly before this probe?” (Possible responses: “on the task” or “off the task”). Whenever participants indicated being “off the task,” a second probe examined their meta-awareness: “How aware were you of where your attention was focused?” (Possible responses: “aware” or “unaware”). Inter-probe intervals varied between 25 s and 35 s and the number of probes (one-fold or two-fold) per run was 25. Three subjects were excluded from the statistical analysis of meta-awareness data. Two subjects were lacking off-task responses during at least one stimulation condition and one subject indicated perfect (i.e., 100%) meta-awareness for all conditions, which appeared unrealistic.

### Auditory stimulation conditions

To examine the effects of monaural beat stimulation, six different stimulation conditions were applied: (1) “headphones only” condition (control 1: *silence*); (2) 220 Hz sine tone (control 2: *sine*; frequency identical to the carrier frequency of the monaural beats); (3) white noise (control 3: *W.N.*); (4) 5 Hz monaural beat (*MB*); (5) 5 Hz monaural beat, applied to the right ear only (*MBri.*); (6) monaural beat with a self-selected frequency (*MBsel.*; choices: 3.5/4/4.5/5/5.5/6/6.5 Hz). All stimuli were applied to both ears, except for stimulus condition 5 (right ear only). Monaural beats with modulation frequencies f_m_ and a carrier frequency of 220 Hz were constructed by superposing two sine waves with the frequencies 220 Hz – f_m_/2 and 220 Hz + f_m_/2. For instance, 5 Hz monaural beats resulted from the superposition of sine waves with frequencies of 217.5 and 222.5 Hz. All auditory stimuli were played using over-ear headphones and with a sound pressure level (SPL) of 60 dB. The SPL was adjusted to 55 dB when participants found the preset volume uncomfortable (this occurred in two cases). Auditory stimulation was played using Psychtoolbox custom code and an integrated MP3 file. All stimulation conditions were created using Tone Generator (NCH software, Canberra, Australia).

### Randomization and counterbalancing procedure

The order of auditory stimulation conditions across the 42 subjects was randomized and counterbalanced in such a way that each of the six stimulation conditions appeared at each position (1,2,…,6) exactly seven times. To accomplish this, consecutive random sequences of the six stimulation conditions were produced (MATLAB random generator; drawing without laying back). Sequences were erased when being identical to a previous sequence or when one of the stimulation conditions occurred more than seven times at a certain position. This procedure was repeated until 42 exactly randomized and counterbalanced sequences were obtained.

### Visual-analog scales

Participants indicated their momentary levels of anxiety, stress and negative mood immediately prior to and after each of the experimental runs (each corresponding to one of the six stimulation conditions) by making tick marks on line-scales. In the same way, their subjective perceptions of the stimulus conditions as distracting, disturbing, uncomfortable, and tiring were also acquired immediately after each of the runs. Minimum, midpoint and maximum of the scales were illustrated by abstract positive (minimum), neutral (midpoint) and negative face ideograms (maximum), as well as by the descriptive titles “very little” (minimum) and “very much” (maximum). The tick marks on the line-scales were then converted to values (to one decimal point) between 0 and 10. For further analysis, all values were divided by the factor 10 and post-run values for anxiety, stress and negative mood were subtracted from pre-run values.

### Experimental measures

Based on experience sampling the following thought-probe measures were analyzed: (1) propensity to mind wander (i.e., proportion of “off-task” responses to first probe); (2) ratio of meta-awareness (i.e., proportion of “aware” responses to second probe; denominator: all “off task” responses). Additionally, based on the SART, the following behavioral measures were evaluated: (3) accuracy of responses to non-targets (reflecting errors of omission); (4) accuracy of responses to targets (reflecting errors of commission); (5) reaction times (for non-targets); (6) standard deviation of reaction times (for non-targets). Moreover, the following measures were derived from the visual-analog scales: (7) Anxiety: post-run minus pre-run; (8) Stress: post-run minus pre-run; (9) Negative mood: post-run minus pre-run; (10) Distracting (perception of the stimulus condition); (11) Disturbing; (12) Uncomfortable; (13) Tiring. Before entering statistical analyses, data for the measures 1–4 and 10–13 were subjected to arcsine transformation to render normal distribution. For the same purpose, data for the measures 7–9 were subjected to Fisher z-transformation.

### Statistical analyses

For each of the measures 1–6 we conducted one-way repeated measure analyses of covariances (ANCOVAs). STIMULATION condition (*silence*, *sine*, *W.N.*, *MB*, *MBri.*, *MBsel.*) was employed as a repeated measure. The combined MW score (MWSCORE) derived from the MW questionnaires (Mrazek et al., 2013; Nakovics et al., 2021) was used as a covariate. In case Mauchly’s test indicated a violation of the assumption of sphericity, *p*-values were Huynh-Feldt-corrected. Least significant difference (LSD) tests were calculated as post-hoc tests. Interactions of STIMULATION*MWSCORE were further analyzed by applying a median split based on MW scores and by conducting separate one-way repeated measure analyses of variance (ANOVAs) for the subgroups with high and low MW scores.

For the measures 7–9, as well as for the measures 10–13 we conducted one-way multivariate analyses of variance (MANOVAs) with STIMULATION condition as a repeated measure. Moreover, for each of the measures 7–13 exploratory one-way repeated measure ANOVAs were evaluated (if necessary, Huynh-Feldt-corrected). Again, STIMULATION condition was employed as a repeated measure and LSD tests were performed as post-hoc tests. Furthermore, Pearson correlation coefficients were calculated for all pairs between the 13 measures based on the average values across all experimental conditions (1–6), as well as based on the average values across the conditions comprising auditory stimulation (2–6).

## Results

### Mind wandering scores

In our sample, the average score of the MWQ was 14.88 ± 3.41 (mean ± s.d.). In the original publication introducing this scale, Mrazek et al. (2013) investigated 663 undergraduate students and reported an average score of 18.86. Thus, the average MWQ score observed in our study is numerically lower than the score observed in Mrazek et al. (2013). The average score of the MEWS was 11.31 ± 6.44 (mean ± s.d.) in our sample. Mowlem et al. (2019) and Nakovics et al. (2021) reported numerically lower average scores for healthy subjects [Mowlem et al. (2019): 4.79 (*N* = 24) and 7.21 (*N* = 29); Nakovics et al. (2021): 7.64 (*N* = 31)], but considerably higher average scores for ADHD patients [Mowlem et al. (2019): 25.00 (*N* = 25) and 27.72 (*N* = 79); Nakovics et al. (2021): 23.77 (*N* = 97)].

### Self-selected beat frequency

To select a modulation frequency, subjects listened to a short sample of seven different recordings of the auditory beats with modulation frequencies ranging from 3.5 Hz to 6.5 Hz (in steps of 0.5 Hz), and were asked to choose what they perceived as the most pleasant stimulus. Participants selected beats with modulation frequencies of 4 Hz (22 times), 3.5 Hz (18 times), and 4.5 Hz (2 times). Modulation frequencies equal to or above 5 Hz were not chosen.

### ANCOVAs (measures 1–6)

**Figure 2:**
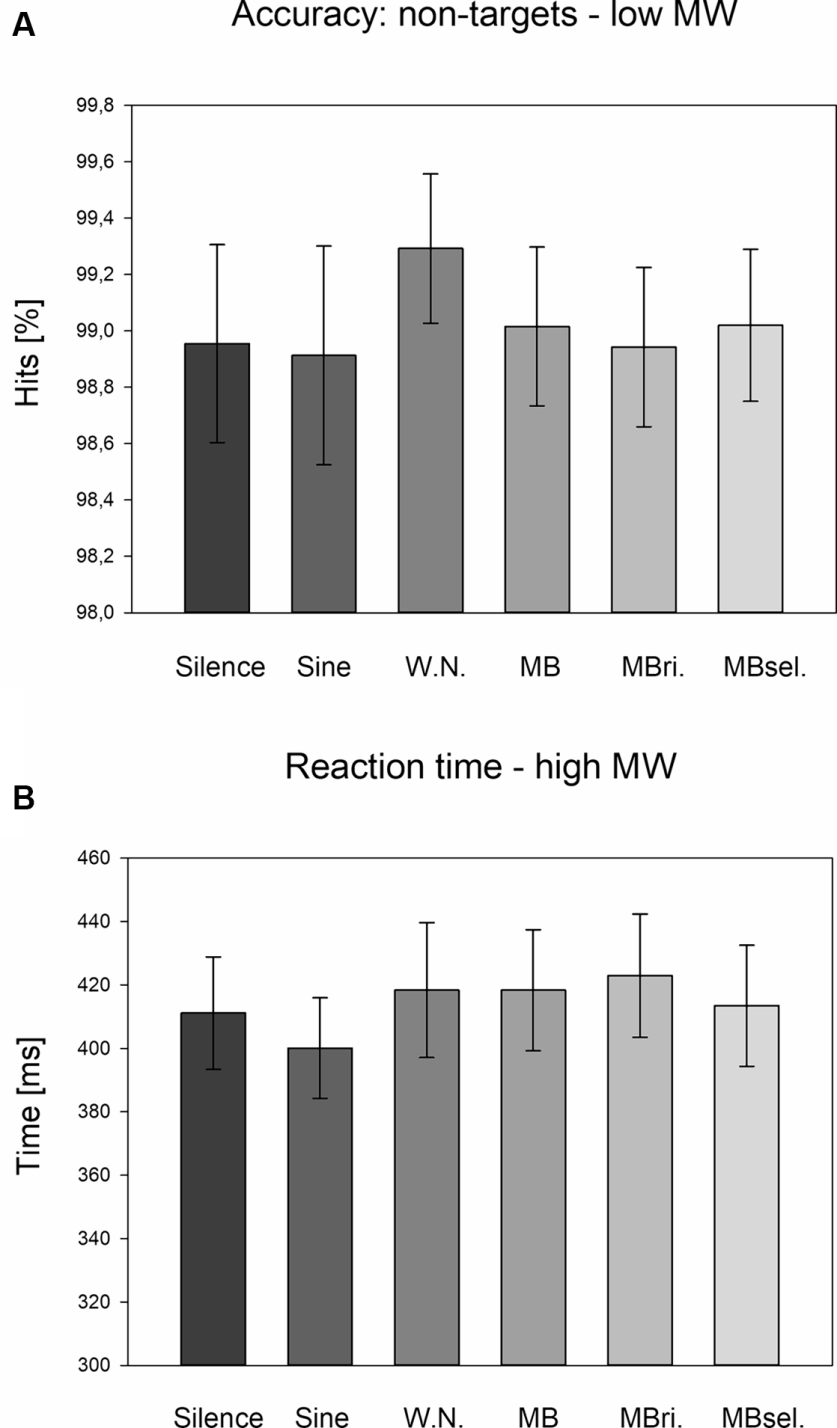
Accuracy for non-targets in case of low MW scores and reaction times in case of high MW scores. **(A)** Average accuracy for non-targets across the subgroup with low MW scores for the six stimulation conditions. Mean and SEM are depicted. Silence, headphones only; Sine, sine wave; W.N., white noise; MB, monaural beat; MBri., monaural beat applied to the right ear; MBsel., self-selected monaural beat. **(B)** Average reaction times across the subgroup with high MW scores for the six stimulation conditions. Mean and SEM are depicted.

For the non-target response accuracy, a significant main effect for STIMULATION (*F*_5,200_ = 2.376, *p* = 0.040, partial eta-squared *η*^2^ = 0.057) and a STIMULATION*MWSCORE interaction (*F*_5,200_ = 2.460, *p* = 0.034, *η*^2^ = 0.057) were observed. Separate ANOVAs for the subgroups with high and low combined MW scores revealed no significant STIMULATION effects. Exploratory post-hoc LSD tests showed higher accuracies during the white noise stimulation condition compared to silence (*p* = 0.030, Cohen’s *d* = 0.35) and self-selected beats (*p* = 0.025, *d* = 0.38) for the subgroup with low MW scores (Figure 2A).

For the reaction times, a trend for a STIMULATION*MWSCORE interaction (*F*_5,200_ = 1.909, *p* = 0.094, *η*^2^ = 0.046) was detected. Separate ANOVAs revealed no significant STIMULATION effects, but almost a trend for the high MW subgroup (*F*_5,100_ = 1.899, *p* = 0.101, *η*^2^ = 0.087). For this subgroup, exploratory post-hoc LSD tests pointed to longer reaction times during 5 Hz beat stimulation to both ears, as well as to the right ear compared to sine wave stimulation (*p* = 0.007, *d* = 0.46 and *p* = 0.002, *d* = 0.56; Figure 2B).

For MW propensity, meta-awareness and the standard deviation of reactions times, no statistically significant main effects or interactions were found.

### MANOVA and ANOVAs (measures 7–9)

The MANOVA for anxiety (post-pre), stress (post-pre), and negative mood (post-pre) indicated a trend for a STIMULATION effect (Wilks’ Lambda = 0.897, *F*_15,561_ = 1.494, *p* = 0.102; Roy’s largest root = 0.089, *F*_5,205_ = 3.650, *p* = 0.003).

**Figure 3:**
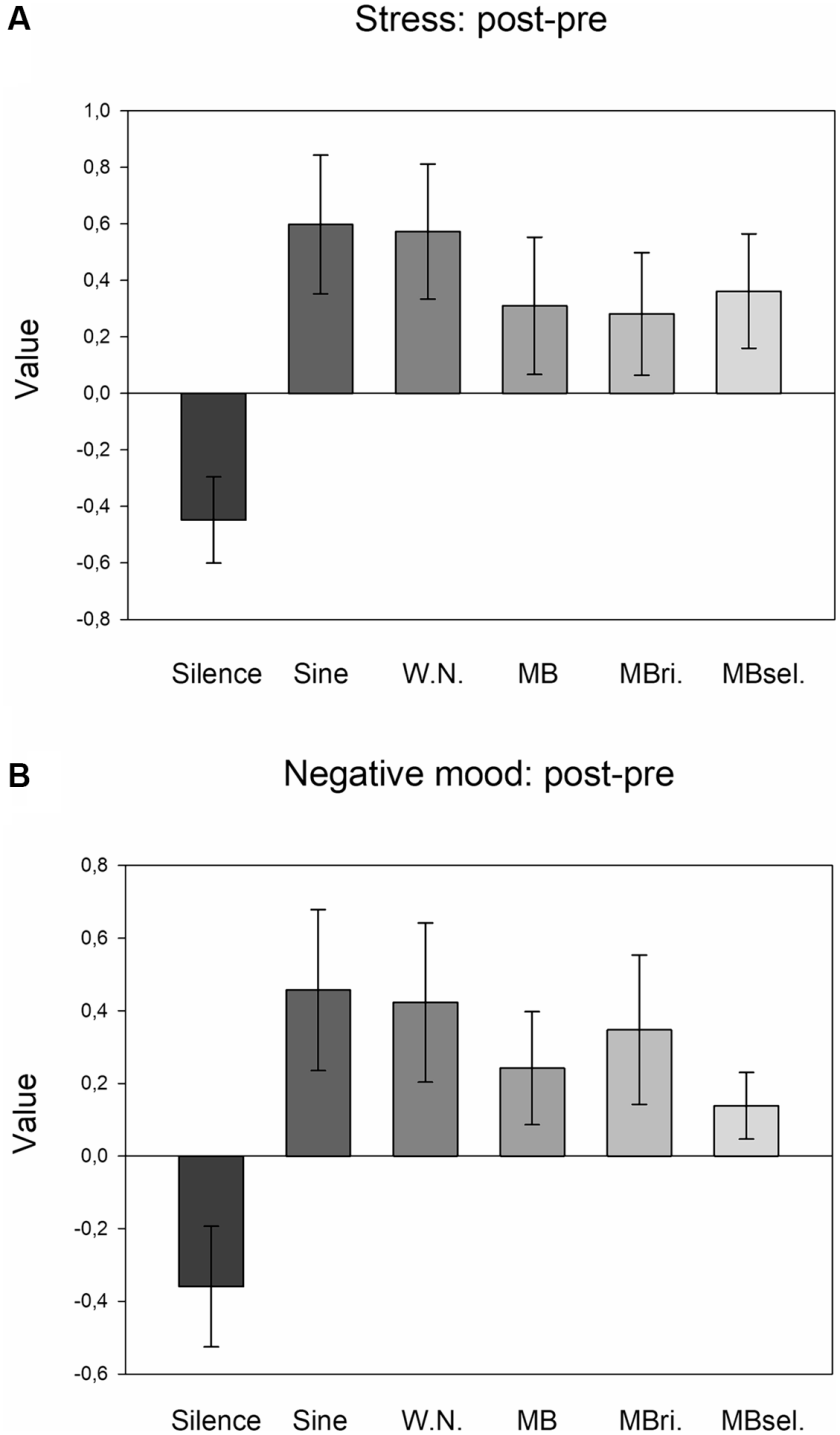
Ratings of stress and negative mood (post-pre). **(A)** Averages of the differences of stress ratings after versus before experimental runs for the six stimulation conditions. Mean and SEM are depicted. **(B)** Averages of the differences of negative mood ratings after versus before experimental runs for the six stimulation conditions. Mean and SEM are depicted.

Exploratory one-way ANOVAs revealed no effect of STIMULATION for anxiety, but significant effects for stress (*F*_5,205_ = 3.246, *p* = 0.011, *η*^2^ = 0.073; Figure 3A), and negative mood (*F*_5,205_ = 2.500, *p* = 0.043, *η*^2^ = 0.058; Figure 3B). For both measures, post-hoc LSD tests pointed to increases of post-versus pre-values in the case of any auditory stimulation (conditions 2–6) compared to silence (each *p* < 0.025, 0.37 ≤ d ≤ 0.59).

### MANOVA and ANOVAs (measures 10–13)

The MANOVA for the indications of distracting, disturbing, uncomfortable and tiring perceptions showed a significant STIMULATION effect (Wilks’ Lambda = 0.511, *F*_20,671_ = 7.515, *p* < 0.001; Roy’s largest root = 0.777, *F*_5,205_ = 31.846, *p* < 0.001).

**Figure 4:**
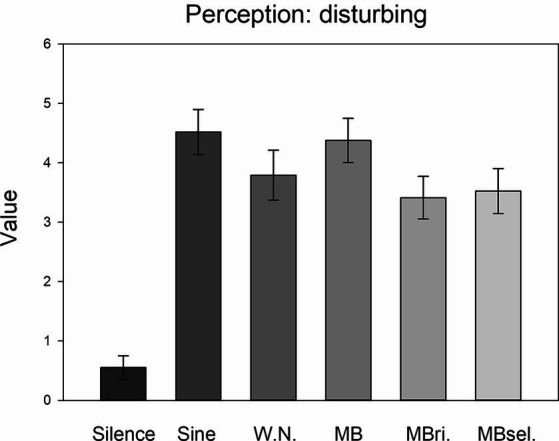
Disturbing perception. Averages of the perception of the stimulation conditions as disturbing for the six conditions. Mean and SEM are depicted.

Exploratory one-way ANOVAs revealed significant STIMULATION effects for each of these perceptions (distracting: *F*_5,205_ = 9.893, *η*^2^ = 0.194; disturbing: *F*_5,205_ = 28.982, *η*^2^ = 0.414 (Figure 4); uncomfortable: *F*_5,205_ = 26.053, *η*^2^ = 0.389; tiring: *F*_5,205_ = 6.551, *η*^2^ = 0.138; each *p* < 0.001). For each perception, post-hoc LSD tests pointed to higher values in the case of any auditory stimulation compared to silence (each *p* ≤ 0.001, 0.55 ≤ d ≤ 1.80). Moreover, sine waves and beats applied to both ears were perceived as more disturbing than auditory beats applied to the right ear only and self-selected beats (each *p* < 0.05, 0.33 ≤ d ≤ 0.35). Furthermore, sine waves were found to be more uncomfortable than white noise, as well as each of the three types of beats (each *p* < 0.005, 0.47 ≤ d ≤ 0.71).

### Pearson-correlations (all measures)

**Figure 5:**
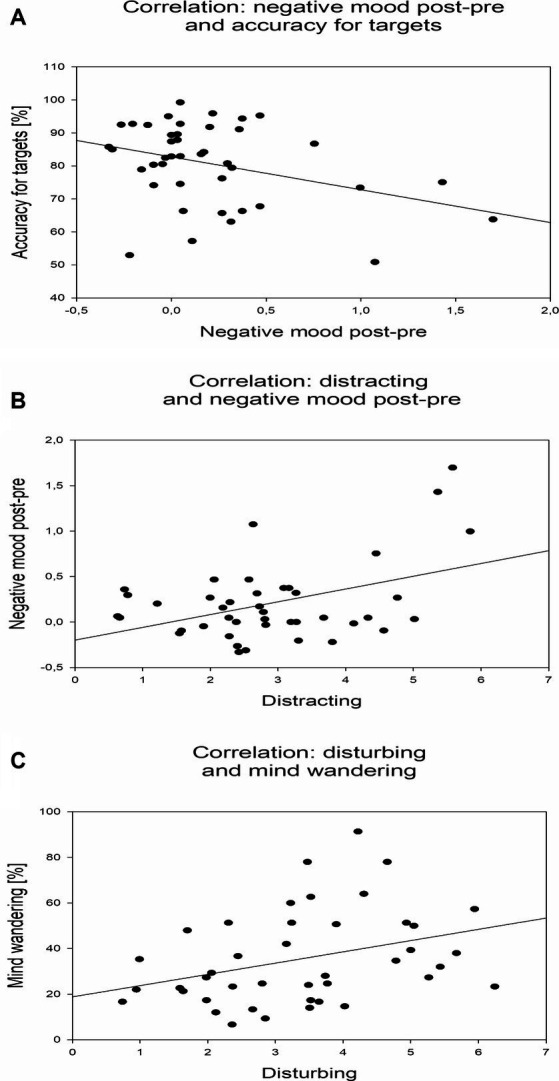
Correlational relationships between experimental measures. **(A)** Dependence of accuracy for targets on negative mood. Individual detection accuracies for targets are depicted across individual differences of negative mood ratings after versus before experimental runs. **(B)** Dependence of negative mood on distracting perception. Individual differences of negative mood ratings after versus before experimental runs are depicted across individual perceptions of the stimulation conditions as distracting. **(C)** Dependence of MW propensity on disturbing perception. Individual MW propensities are depicted across individual perceptions of the stimulation conditions as disturbing.

Table 1 lists basic psychometric qualities of the experimental variables (see Schumann et al., 2022b) in terms of skewness and Cronbach’s alpha (across conditions 1–6 and 2–6). Table 2 shows a summary of Pearson-correlations between the thirteen different measures (averages across conditions 1–6; statistically significant correlations are marked with asterisks). Regarding the behavioral SART measures, reaction times and standard deviations of reaction times were negatively correlated with the accuracy for non-targets (*r* = −0.641; *r* = −0.811). However, reaction times were also positively correlated with the accuracy for targets (*r* = 0.635). Negative mood exhibited a positive correlation with stress (*r* = 0.446), and a negative correlation with the accuracy for targets (*r* = −0.310; Figure 5A). Distracting and disturbing perceptions of the stimulation conditions predicted negative mood (*r* = 0.380; Figure 5B; *r* = 0.321). Moreover, disturbing perception was positively correlated with MW propensity (*r* = 0.343, Figure 5C).

**Table 1:** Psychometric qualities of the experimental variables.

	Skewness	Reliability conditions 1–6	Reliability conditions 2–6
MW	0.620	0.912	0.898
MA	−0.234	0.828	0.828
NT_acc	−0.844	0.893	0.876
T_acc	−0.261	0.910	0.905
RT	1.457	0.981	0.978
SD_RT	0.212	0.954	0.946
Anxiety (before)	1.088	0.955	0.947
Anxiety (after)	0.895	0.955	0.948
Stress (before)	0.346	0.889	0.866
Stress (after)	0.489	0.888	0.877
Mood (before)	0.616	0.924	0.924
Mood (after)	0.923	0.883	0.860
Distractive	0.152	0.600	0.692
Disturbing	0.024	0.642	0.695
Uncomfortable	0.050	0.618	0.674
Tiring	−0.110	0.717	0.761

Skewness and reliablity in terms of Cronbach’s alpha across conditions 1–6 and conditions 2–6 are listed for the experimental variables.

**Table 2:** Summary table of Pearson-correlations (based on conditions 1–6).

	MW	MA	NT_acc	T_acc	RT	SD_RT	Anxiety	Stress	Mood	Distract.	Disturb.	Uncomf.	Tiring
MW													
MA	0.183												
NT_acc	−0.025	−0.133											
T_acc	−0.011	0.267	−0.058										
RT	0.223	0.317 #	−0.641 ***	0.635 ***									
SD_RT	0.255	0.262	−0.811 ***	0.220	0.742 ***								
Anxiety	−0.031	0.094	0.072	0.013	0.036	−0.045							
Stress	−0.041	−0.088	0.253	−0.185	−0.191	−0.205	0.257 #						
Mood	0.139	0.051	−0.127	−0.310 *	−0.014	0.205	0.048	0.446 **					
Distract.	0.160	−0.115	−0.125	−0.049	0.220	0.256	0.143	0.206	0.380 *				
Disturb.	0.343 *	0.002	0.023	−0.263 #	−0.079	0.028	0.126	0.290 #	0.321 *	0.657 ***			
Uncomf.	0.288 #	−0.110	0.027	−0.154	−0.080	0.018	−0.040	0.170	0.246	0.533 ***	0.892 ***		
Tiring	0.113	−0.017	0.032	−0.088	−0.015	0.092	−0.054	−0.071	0.037	0.306 *	0.339 *	0.259 #	

Pearson-correlation coefficients between the 13 measures (average values across conditions 1–6) are depicted. Significance values (two-tailed) are coded as following: ***p < 0.001; **p < 0.01; *p < 0.05; #*p* ≤ 0.1. MW, mind wandering; MA, meta-awareness; NT_acc, accuracy for non-targets; T_acc, accuracy for targets; RT, reactions times; SD_RT, standard deviation of reaction times; Distract., distracting; Disturb., disturbing; Uncomf., uncomfortable.

Pearson-correlations between the thirteen measures based on averages across conditions 2–6 (i.e., the conditions comprising auditory stimulation) are shown in Table 3. Overall, the correlation pattern appears similar to the pattern based on averages across all conditions (Table 1), with a few additional significant correlations and trends. Most notably, the perception of discomfort (uncomfortable) also now exhibits a significant positive correlation with negative mood (*r* = 0.341). Moreover, the positive correlation between disturbing perception and stress shifted from a trend to significance (*r* = 0.376).

**Table 3:** Summary table of Pearson-correlations (based on conditions 2–6).

	MW	MA	NT_acc	T_acc	RT	SD_RT	Anxiety	Stress	Mood	Distract.	Disturb.	Uncomf.	Tiring
MW													
MA	0.206												
NT_acc	−0.015	−0.130											
T_acc	−0.022	0.280 #	−0.038										
RT	0.198	0.317 #	−0.631 ***	0.621 ***									
SD_RT	0.260 #	0.263	−0.830 ***	0.189	0.728 ***								
Anxiety	0.025	0.121	0.111	0.021	0.005	−0.076							
Stress	0.022	−0.059	0.244	−0.220	−0.212	−0.183	0.276 #						
Mood	0.167	0.082	−0.133	−0.380 *	−0.038	0.171	0.008	0.541 ***					
Distract.	0.149	−0.040	−0.095	−0.060	0.184	0.163	0.249	0.263 #	0.412 **				
Disturb.	0.307 *	0.031	0.047	−0.216	−0.082	−0.019	0.233	0.376 *	0.401 **	0.711 ***			
Uncomf.	0.260 #	−0.077	0.004	−0.120	−0.055	0.032	0.105	0.255	0.341 *	0.615 ***	0.898 ***		
Tiring	0.167	−0.021	0.045	−0.047	0.10	0.069	0.130	0.037	0.075	0.383 *	0.437 **	0.371 *	

Pearson-correlation coefficients between the 13 measures (average values across conditions 2–6) are depicted. Significance values (two-tailed) are coded as following: ***p < 0.001; **p < 0.01; *p < 0.05; #*p* ≤ 0.1.

## Discussion

The main aim of the present study was to elucidate the individual perception of different stimulation conditions and to understand how this perception is linked to changes in subjective states, behavioral SART measures and MW. In accordance with our assumption, we found that any auditory stimulation applied during the SART was perceived as more distracting, disturbing, uncomfortable and tiring than silence. As a side note, beats applied to the right ear and self-selected beats were experienced as being less disturbing than 5 Hz beats applied to both ears. Interestingly, all participants chose beat frequencies below 5 Hz, most often 4 Hz, for the self-selected condition. Furthermore, post-versus pre-task increases of stress and negative mood were more pronounced for any auditory stimulation compared to silence. Since stress and negative mood actually decreased during silence (see Figure 3; paired T-Tests, each *p* < 0.05), the auditory stimulation-related increases cannot be attributed to performance of the attentional task *per se*.

Importantly, correlation analyses revealed that perception of the auditory conditions as disturbing was directly related to MW propensity. Moreover, distracting, disturbing and uncomfortable perceptions predicted negative mood. This result is reminiscent of the experimental induction of negative and positive moods by applying different sounds (Bradley and Lang, 2000; Stevenson and James, 2008). In turn, negative mood was inversely correlated with response accuracy for targets, a behavioral measure often regarded as an indicator of MW (Helton et al., 2009). In other words, negative mood led to more errors of commission. This finding is reminiscent of studies reporting more SART errors of commission after experimental induction of negative versus positive mood using video clips (Smallwood et al., 2009), as well as for groups with high versus low Beck Depression Index scores (Murphy et al., 2013). Accordingly, our data suggest that the adverse perception of auditory stimulation can influence MW and attentional performance, and that this influence may be mediated by changes in mood.

Concerning the measurement of anxiety, stress, mood and the perceptions of the auditory conditions using visual-analog scales, it is debatable at which time points these scales should be best presented. We chose to present these scales immediately (before and) after each of the experimental runs (each corresponding to one of the six stimulation conditions). An alternative approach would have been to present these scales (before and) after the entire experiment. One advantage of this approach would be that subjects could compare conditions against each other after completing the experiment. However, a significant disadvantage would be the potential for fading memory of the feelings and perceptions of the stimulation conditions. Moreover, it has been demonstrated that mood often severely declines and mental fatigue often increases across cognitive experiments (Schumann et al., 2022a; Jangraw et al., 2023), suggesting that more frequent measurements may be preferable. On the other hand, with the chosen option, it is not clearly defined against which condition participants should compare their assessments. One way to address this uncertainty could be to compare feelings and perceptions against a standard. For example, an experimental run employing a standard stimulation condition (e.g., pink noise) could be added at the beginning of the experiment. However, since this condition would always be the first and would not be included in the balancing scheme, it would need to be excluded from further analysis.

Regarding behavioral SART performance, we observed evidence for fewer errors of omission during white noise stimulation compared to silence and self-selected beats in the case of low MW scores. This result is in line with findings suggesting facilitating effects of white stimulation on attentional and cognitive performance (e.g., Angwin et al., 2017; Awada et al., 2022). Moreover, we found tentative evidence for increased reaction times during 5 Hz beats compared to sine wave stimulation in the case of high MW scores. Since reaction times were negatively correlated with non-target accuracy, but positively correlated with target-accuracy (see Tables 1, 2), this effect could be interpreted as being potentially related to both decreases and increases in SART performance.

We would like to mention that the implemented version of the SART does not actively address errors of omission because of the low target probability. It has been demonstrated that as the SART target probability decreases, errors of commission decrease while errors of commission increase (e.g., Bedi et al., 2023). Consequently, participants in the implemented version of the SART are more susceptible to errors of commission than to errors of ommission. Moreover, error rates and reaction times may by influenced by the inter-stimulus interval employed, with arousal levels potentially mediating this relationship. Specifically, shorter intervals may increase arousal levels, while longer intervals may decrease them (e.g., Steinborn and Langner, 2012).

While we found evidence for a modulation of MW propensity due to monaural beat stimulation in our previous studies (Chaieb et al., 2020, 2022b), we did not observe such an effect in our current study. Differences in study design and methodology might account for this discrepancy. For instance, gender (female and male) subgroups were equally sized by design in Chaieb et al. (2020), and the decrease of MW propensity for monaural 5 Hz beats compared to control conditions was most pronounced in the male subgroup. However, most participants identified as female in the present study. In Chaieb et al. (2022b) subjects were preselected for high scores of the MW questionnaire (indicating high trait MW) (Mrazek et al., 2013). Moreover, modulation rates of the monaural beat stimuli were continuously shifted between 4 and 8 Hz to render the beat stimuli less monotonous. Furthermore, the lengths of SART blocks were shorter in the present study (around 15 min) than in Chaieb et al. (2020; around 30 min) and Chaieb et al. (2022b; around 35 min), which may also have affected the outcome. In this context, we recently reported that the temporal evolution of MW characteristics during SART blocks may depend on auditory stimulation conditions (Chaieb and Fell, 2023).

In conclusion, the present study investigated the effects of auditory beat stimulation and control conditions on MW, as assessed by experience sampling, as well as on SART performance measures, and subjective feelings. Changes in anxiety, stress and negative mood, as well as the subjective perception of the stimulation conditions were measured via visual-analog scales. Our data showed that different types of auditory stimulation during attentional task execution were perceived as more distracting, disturbing, uncomfortable and tiring than silence and resulted in enhanced stress and negative mood. Adverse perception of auditory stimulation was related to increases of MW propensity and negative mood, and the latter, in turn, was linked to a deterioration of SART performance. In this sense, it is possible that adverse perception of auditory beat stimuli may counteract a potentially effective neurophysiologically mediated reduction of MW.

As a consequence, approaches aiming at utilizing auditory stimulation and, in particular, beat stimulation for the modulation of MW should try to optimize stimulation with regard to pleasant perception, or at least mediate adverse perceptual side effects. The present data, for instance, indicate that relatively low modulation frequencies may be favored. Moreover, lower sound pressure levels may be advantageous, as has recently been demonstrated for attentional performance under white noise stimulation with 45 dB compared to 65 dB (Awada et al., 2022). Furthermore, auditory beats may be embedded into more pleasant stimuli such as music (Liu et al., 2022) or nature sounds (Munro and Searchfield, 2019) to facilitate positive perception. Finally, continuous shifts of either the carrier frequency (Chaieb et al., 2017) or the modulation frequency (Chaieb et al., 2022b) may render auditory beats less monotonous and more versatile.

Taken together, our results suggest that silence may be preferable to any kind of acoustical stimulation concerning MW, mood, and performance during mental work. However, it should be noted that our data pertain to healthy subjects with relatively low MW scores. Future research could explore the extent to which these findings depend on the overall level of MW, on personality traits such as extraversion and introversion (e.g., Moradi et al., 2018), and on clinical diagnoses or symptoms like depression, anxiety and ADHD (e.g., Nigg et al., 2024). Another underexplored issue is the frequent lack of consistent alignment between subjective MW, as assessed by experience sampling, and objective performance measures related to MW (Schumann et al., 2022a). Moreover, research on the fluctuations of MW and mood on shorter time scales has recently gained increasing interest (e.g., Jangraw et al., 2023; Zanesco et al., 2024). Future investigations could clarify whether the detrimental effects of auditory stimulation on MW and mood observed in this study are solely associated with attentional performance or also extend to resting phases and everyday activities.

## Data availability statement

The raw data supporting the conclusions of this article will be made available by the authors, without undue reservation.

## Ethics statement

The studies involving humans were approved by the Ethics Committee of the Medical Faculty of the University of Bonn. The studies were conducted in accordance with the local legislation and institutional requirements. The participants provided their written informed consent to participate in this study.

## Author contributions

LS: Data curation, Formal analysis, Investigation, Writing – original draft, Writing – review & editing. LC: Data curation, Formal analysis, Methodology, Writing – review & editing. MD: Methodology, Writing – review & editing. TR: Formal analysis, Methodology, Writing – review & editing. JF: Conceptualization, Formal analysis, Funding acquisition, Methodology, Supervision, Writing – original draft, Writing – review & editing.
